# Clinical Society

**Published:** 1897-04-17

**Authors:** 


### CLINICAL SOCIETY.

Mr. Howard Marsh read a paper on a case of Sarcoma of the Prostate Gland. He said that this disease might occur at any period of life from infancy to old age. The duration of the disease was variable. In the case related it was probably between four and five years. Bilroth had mentioned a case of "cancer" which had lasted six years; while Spanton, on the other hand, recorded one in which death took place 13 weeks after interference with micturition was noticed. He thought that probably this great variation in the duration of the disease had to do with the structure of the tumour, which might, as in his case, be mostly formed of spindle-shaped elements, or might be of a more rapidly-growing type.

Mr. Bland Sutton spoke of the chaotic state of ordinary surgical ideas on this subject, which must persist so long as surgeons attempted to divide malignant growths in other organs, even in the prostate, according to ideas gained from study of the diseases of the breast, *viz.*, into scirrhous and encephaloid. He stated that true carcinoma did attack the prostate, and took on the typical course of a cancerous growth, producing secondary deposits in the bones. Such disease he looked on as a malady of advanced life. The disease which occurred in earlier life was sarcomatous. This might occur in quite young children. The tendency to it lasted through early life, and then there was a period of almost immunity, lasting, perhaps, a score of years, and then an age arrived when sarcomata were again liable to occur. This peculiarity occurred in other organs, notably the kidney, eyeball, and ovary.

Mr. Shattock suggested that, in view of the influence which removal of the testicles had over hypertrophy of the prostate, and that which removal of the ovaries also seemed to exert over the progress of cancer of the breast, it might be well to remove the testicles in these cases of malignant growth in the prostate, as a means, at least, of retarding development.

On the other hand, it was pointed out in the discussion that in children, at any rate, some of these growths arose, not from the prostate itself, but from the cellular tissue outside—the recto-vesical cellular tissue—the tumour being of a myxo-sarcomatous type.

Mr. Buckston Browne said that urethral hæmorrhage was not common in sarcoma, but often occurred in carcinoma, and was in many cases precipitated by the use of the catheter. He also drew attention to the neuralgic pains running down the sciatic nerves which often accompanied the disease.

Mr. Raymond Johnson read a paper on a "Multilocular Ovarian Cyst in a Child aged five years; Axial Rotation; Ovariotomy." The tumour extended high into the abdomen, but could not be outlined below where it extended into the pelvis. It did not extend into the loin. The abdominal enlargement was dull on percussion and very tender, and was thought to be an abscess. On incising the abdominal walls, however, and opening the peritoneal cavity, a smooth walled cyst of the left ovary was found, the pedicle of which had undergone two complete rotations. The pedicle was ligatured, the tumour removed, and the patient made a complete recovery. On examination of the growth it was found to be a multilocular ovarian cyst, which was in some parts distended by hæmorrhage, separating the bundles of fibrous tissue. On referring to the literature of the subject, he had found that the majority of ovarian tumours in young children were dermoid cysts.

Mr. Bland Sutton said that about a fourth of the ovarian tumours met with in children were sarcomata. Ovariotomy in children between ten and fifteen years of age was perhaps more successful than at any other period of life. Below ten, however, it was not so generally successful. Cases of tumour under five years of age, of such a size as to be clinically discoverable, were rare. Rotation in adults was commonly caused either by pregnancy, which was the great cause, or by the presence of a fibroid. The condition, however, was also common in children.

Mr. W. G. Spencer related the history of a lady aged 20 who had had hydatid cysts removed from the left pleura, from behind the mesentery, and from the right lobe of the liver. The case had at first appeared like one of empyema on the left side, pointing towards the umbilicus, but on making an incision through the left rectus muscle clear colourless fluid escaped at a high pressure. The nozzle of an irrigator was inserted and the parasitic cyst of hydatid was floated out. It was then found that notwithstanding the position of the incision the cavity which it had entered was situated above the diaphragm. On the patient partially coming round from the anæsthetic (ether) she gave a cough and immediately the compressed lung expanded and filled the left side of the chest.

After this operation the general health improved and the physical signs in the left chest became normal, but the liver was found to be enlarging. It ultimately reached from the level of the nipple down to the umbilicus, but there was no circumscribed tumour. Three months after the first operation another was performed, the incision this time being through the right rectus. The surface of the liver appeared to be normal, but a tense cyst was found deep down behind it covered by intestines and mesentery. The latter was divided and the cyst was tapped, drawn forwards, and opened, the parasitic cyst being floated out by irrigation as on the other side. The right lobe of the liver, however, still appeared large, and there seemed to be a tense swelling in its hinder part above the kidney, although there was no projecting tumour. The cyst in this case was evidently very deeply placed and lay near the portal fissure. Mr. Spencer, therefore, sutured the abdominal wound and then turned the patient over and made an incision upon the tenth rib in the right axillary line, opened the pleura, which was not adherent, stitched the diaphragm to the upper edges of the pleural wound and then cut through it, entering the abdominal cavity and exposing the upper and hinder surface of the liver above the kidney. The liver here was tense and a small trocar being inserted reached the cyst. The opening thus made was enlarged and a gauze drain was inserted, the cavity being too deeply placed to attempt the removal of the parasitic cyst in the then condition of the patient. During the process of recovery, which was ultimately complete, a large amount of fluid and of shreds of cyst escaped by the drainage wound. Mr. Spencer thought that the case showed the great advantages of incision over aspiration. If the first cyst had been aspirated the patient would have been exposed to the danger of sudden death which has so often happened on tapping hydatids in the thorax. Any attempt to tap the second cyst would have involved much risk of puncture of the intestines or of some of the large veins with which the mesentery was crowded, while as to the third it was dangerously deep, and its tension was so great that if it had been tapped through the pleura, there would have been much risk of its contents escaping into that cavity and leading to the same dangers as attend the tapping of a pleural cyst. The patient is now in perfectly good health, and a day or two before the meeting told Mr. Spencer that she had been dancing.

According to Australian writers, the aspiration of hydatid cysts is directly fatal in at least 18 per cent. Of the rest at least 50 per cent. are not cured. Owing to improvements in the methods of operation the treatment by incision is most successful in all early and uncomplicated cases. Only a small wound is required through which the parasitic cyst is most easily removed, and the thin walled adventitious cyst falls together, and is united without drainage whenever possible. The longer the hydatid cyst remains the more adherent becomes the parasitic cyst, and the thicker the wall of the adventitious cyst, so that the former is removed with difficulty, the latter does not collapse readily, and hence prolonged drainage is necessary with all its attendant troubles. The suppuration or rupture of the hydatid immediately puts the patient's life in grave danger.

